# Supplementary material for: Systems Analysis of Protein Fatty Acylation in Herpes Simplex Virus-Infected Cells Using Chemical Proteomics
Source: Chem Biol. 2015 Aug 20;22(8):1008–17. doi: 10.1016/j.chembiol.2015.06.024 (PMC4543063; doi:10.1016/j.chembiol.2015.06.024)
Supplement: Document S1. Figures S1–S3 [file mmc1.pdf]

Chemistry & Biology, Volume *22*

Supplemental Information

Systems Analysis of Protein Fatty Acylation in Herpes Simplex Virus-Infected Cells Using Chemical Proteomics

Remigiusz A. Serwa, Fernando Abaitua, Eberhard Krause, Edward W. Tate, and Peter O'Hare

Supplemental Information

Supplemental Figures and Tables

Supplemental Figures

Figure S1: Metabolic tagging in uninfected RPE-1 cells with fatty acid probes (YnMyr and YnPal). Relates to Figures 2.

Figure S2: HSV infection-dependent relative palmitoylation levels cross-compared to Chx treatment-dependent relative palmitoylation levels. Relates to SI Table 6.

Figure S3: Detection of HSV 1 [17] (MOI = 5) proteins metabolically tagged in RPE-1 cells with YnPal and YnMyr. Relates to SI Table 7.

Supplemental Tables

Supplementary tables have been incorporated in separate Microsoft Excel documents.

SI Table 1 HSV induced changes to protein palmitoyltion. List of 1292 proteins quantified in lysates obtained from metabolic tagging with YnPal, ligation to AzTB, and affinity enrichment. Relates to Figure 3.

SI Table 2 HSV induced changes to protein myristoylation. List of 208 proteins containing N-terminal glycine (myristoylation motif) quantified in lysates obtained from metabolic tagging with YnMyr and affinity enrichment. Relates to Figure 3.

SI Table 3 HSV induced changes to total protein abundances. List of 1428 proteins quantified in lysates obtained from metabolic tagging experiments (with YnMyr and YnPal) prior to affinity enrichment. Relates to Figure 3.

SI Table 4 HSV induced changes to nascent protein abundances. List of 510 proteins quantified in lysates obtained from 'Pulse SILAC' metabolic tagging experiments (with YnMyr) prior to affinity enrichment. At the bottom is given a list of 15 protein containing myristoylation motif (N-terminal glycine) quantified in lysates obtained from 'Pulse SILAC' metabolic YnMyr tagging experiments followed by ligation to AzTB and affinity enrichment. Relates to Figure 4.

SI Table 5 Chx induced changes to protein palmitoylation and total protein abundances. List of 496 proteins quantified from metabolic tagging with YnPal in the presence or absence of cycloheximide after ligation to AzTB, and affinity enrichment (palmitoylated). Relates to SI Figure 2.

SI Table 6 Cross comparison of the effects of HSV and Chx on protein palmitoylation. HSV infection-dependent relative palmitoylation levels correlated to Chx treatment-dependent relative palmitoylation levels in a 2-sample test. Relates to SI Figure 2.

SI Table 7 Fatty acylation of HSV encoded proteins. List of 56 HSV encoded proteins quantified in lysates obtained from metabolic tagging with YnMyr/Myr and YnPal/Pal probe pairs following ligation to AzTB and affinity enrichment. Relates to Figure 5.

SI Table 8 YnMyr tagging of HSV proteins in response to chemical inhibition of myristoylation. List of 9 HSV proteins that possess N-myristoylation motif (N-terminal glycine) quantified in lysates obtained from metabolic tagging with YnMyr in the presence and absence of an N-myristoyltransferase inhibitor. Relates to Figure 5.

SI Table 9 Functional annotation chart for proteins with decreased levels of palmitoylation. Results of functional annotation analysis performed using David Bioinformatics Resource on the set of 116 human proteins. Relates to Figure 3b and SI Table1.

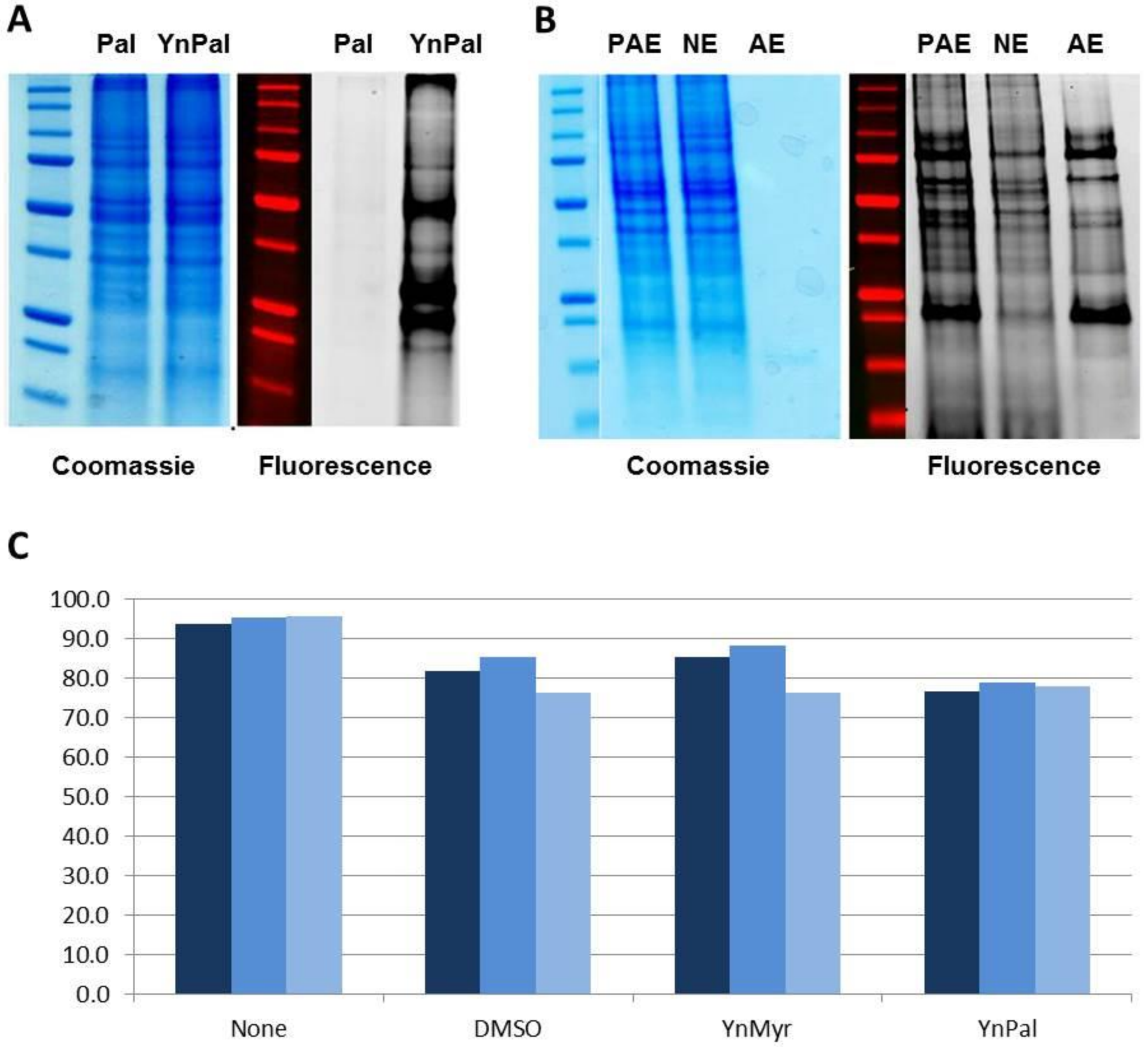

SI Figure 1. Metabolic tagging in uninfected RPE-1 cells with fatty acid probes (YnMyr and YnPal). A. RPE-1 cells were incubated with YnPal (25 µM) or with palmitic acid, Pal (25 µM) for 24 h. Cells were lysed, proteins in lysates reacted with AzTB and resolved by SDS-PAGE. Fluorescence of the gel was recorder prior to Coomassie staining (loading control). B. RPE-1 cells were incubated with YnMyr (25 µM) for 24 h. Cells were lysed, proteins reacted with AzTB and affinity enriched (streptavidin beads). Three protein fractions were applied to SDS-PAGE: AzTB reacted proteins prior to affinity enrichment (PAE); affinity enrichment (AE) and non-enriched (NE). Fluorescence of the gel was recorder prior to Coomassie staining. C. RPE-1 cells were incubated in the absence of additives (None) or in the presence of DMSO vehicle (0.1% v/v), YnMyr (25 µM), and YnPal (25 µM) for 24 h followed by viable cell counting using an automated cell counter (Countess by Invitrogen). Shades of blue indicate experimental series (n = 3).

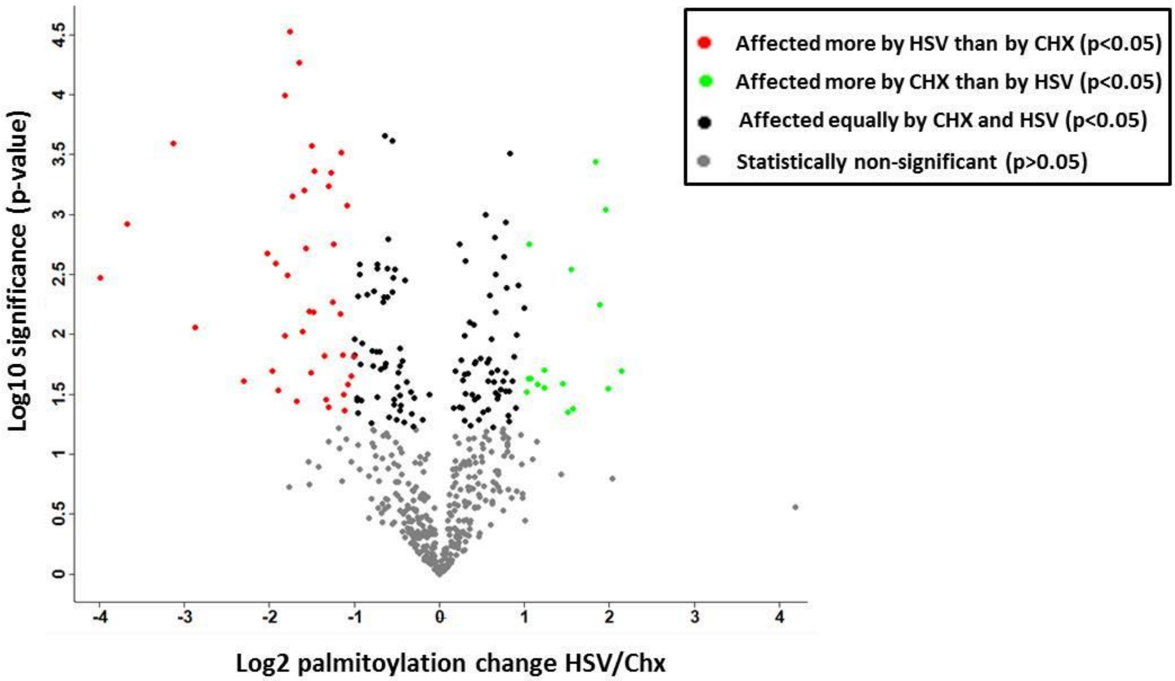

SI Figure 2. HSV infection-dependent relative palmitoylation levels cross-compared to Chx treatment-dependent relative palmitoylation levels (two-sample test, permutation based FDR = 0.05). Color-coded are different classes of proteins labelled with YnPal: proteins of which palmitoylation is affected more by HSV (red); proteins of which palmitoylation is affected more by Chx (green); proteins of which palmitoylation is affected to similar extent by HSV and Chx (black); proteins which could not be unequivocally included into the aforementioned groups due to relatively large variance within at least one of the experimental groups tested (grey). Protein identities are given in SI Table 6.

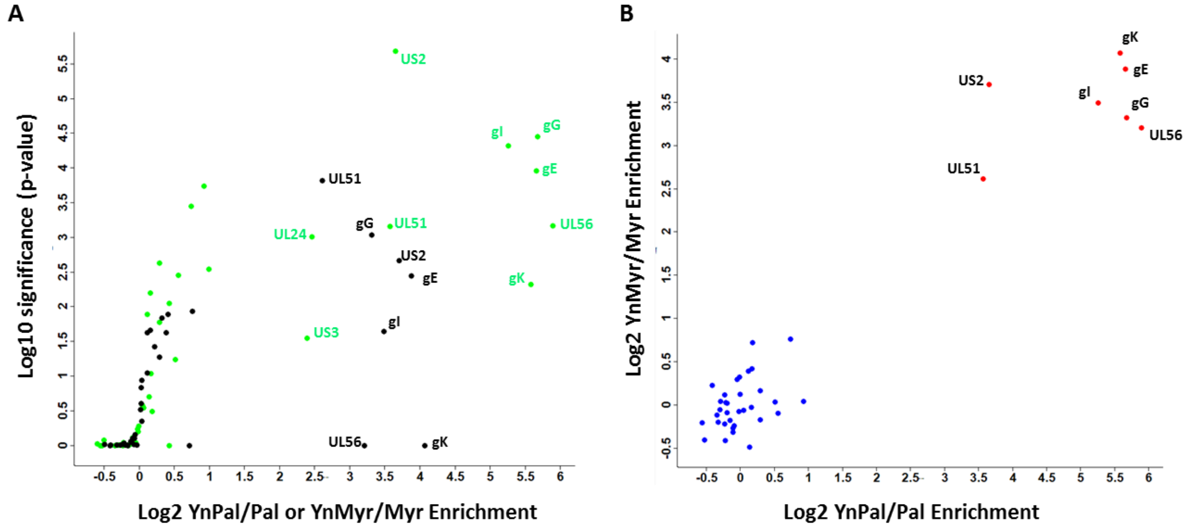

SI Figure 3. Detection of HSV 1 [17] (MOI = 5) proteins metabolically tagged in RPE-1 cells with YnPal and YnMyr. A. Palmitoylated (green) and Myristoylated (black) proteins identified on the basis of the relative enrichment from tagging with YnPal/Pal (5-19 hpi) and YnMyr/Myr (5-9 hpi), respectively. Enrichment (x-axis) plotted against significance (y-axis). B. Overlap of average YnPal/Pal and YnMyr/Myr enrichment values. Lipidated HSV protein candidates detected in both analyses are indicated in red amongst non-lipidated HSV proteins (blue).
