# Supplementary material for: Systems Analysis of Protein Fatty Acylation in Herpes Simplex Virus-Infected Cells Using Chemical Proteomics
Source: Chem Biol. 2015 Aug 20;22(8):1008–17. doi: 10.1016/j.chembiol.2015.06.024 (PMC4543063; doi:10.1016/j.chembiol.2015.06.024)
Supplement: Document S2. Article plus Supplemental Information [file mmc11.pdf]

Article

Chemistry & Biology

Systems Analysis of Protein Fatty Acylation in Herpes Simplex Virus-Infected Cells Using Chemical Proteomics

Graphical Abstract

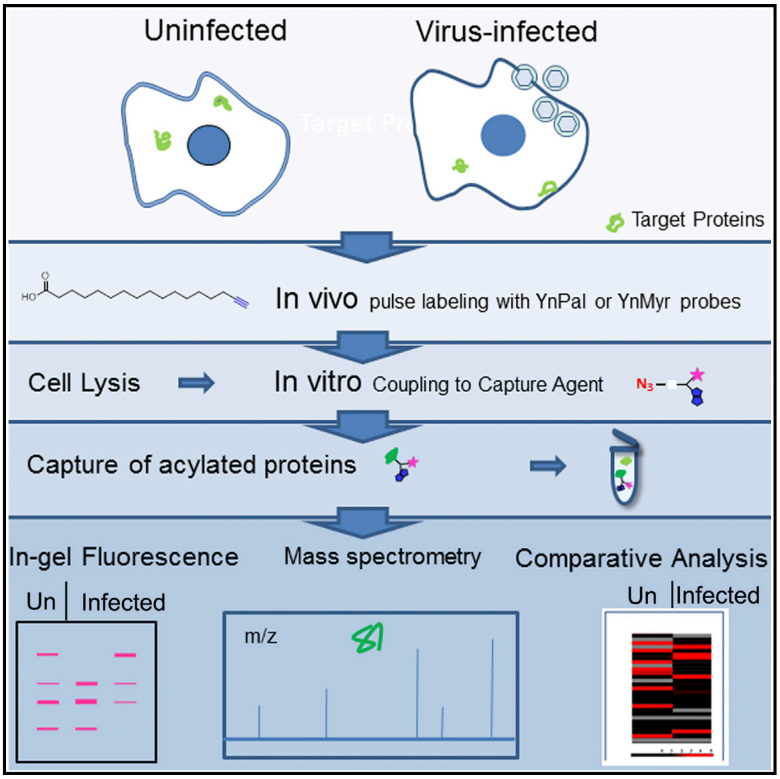

Authors

Remigiusz A. Serwa, Fernando Abaitua, Eberhard Krause, Edward W. Tate, Peter O'Hare

Correspondence

pohare@imperial.ac.uk (P.O.),
e.tate@imperial.ac.uk (E.W.T.)

In Brief

Serwa et al. develop a roadmap for the systematic investigation of protein acylation during infection, exemplified by analysis of a large complex DNA virus, herpes simplex virus. Using bio-orthogonal probes and capture reagents, the authors quantify alterations to host acylation and identify novel virus-encoded acylated proteins.

Highlights

- High-throughput analysis of protein acylation during herpes virus infection described
- Host myristoylation is reduced globally while palmitoylation is affected selectively
- Specific host proteins whose palmitoylation is selectively repressed are identified
- New acylated viral proteins are identified with crucial functions during replication

Serwa et al., 2015, Chemistry & Biology *22*, 1008–1017
August 20, 2015 ©2015 The Authors
http://dx.doi.org/10.1016/j.chembiol.2015.06.024

Chemistry & Biology
Article

Systems Analysis of Protein Fatty Acylation in Herpes Simplex Virus-Infected Cells Using Chemical Proteomics

Remigiusz A. Serwa,[1] Fernando Abaitua,[2] Eberhard Krause,[3] Edward W. Tate,[1,*] and Peter O'Hare[2,*]
[1]Department of Chemistry, Imperial College London, Exhibition Road, London SW7 2AZ, UK
[2]Section of Virology, Faculty of Medicine, Imperial College London, Norfolk Place, London W2 1QN, UK
[3]Leibniz-Institut für Molekulare Pharmakologie (FMP), Robert-Rössle Street 10, 13125 Berlin, Germany
*Correspondence: pohare@imperial.ac.uk (P.O.), e.tate@imperial.ac.uk (E.W.T.)
http://dx.doi.org/10.1016/j.chembiol.2015.06.024
This is an open access article under the CC BY license (http://creativecommons.org/licenses/by/4.0/).

SUMMARY

Protein fatty acylation regulates diverse aspects of cellular function and organization and plays a key role in host immune responses to infection. Acylation also modulates the function and localization of virus-encoded proteins. Here, we employ chemical proteomics tools, bio-orthogonal probes, and capture reagents to study myristoylation and palmitoylation during infection with herpes simplex virus (HSV). Using in-gel fluorescence imaging and quantitative mass spectrometry, we demonstrate a generalized reduction in myristoylation of host proteins, whereas palmitoylation of host proteins, including regulators of interferon and tetraspanin family proteins, was selectively repressed. Furthermore, we found that a significant fraction of the viral proteome undergoes palmitoylation; we identified a number of virus membrane glycoproteins, structural proteins, and kinases. Taken together, our results provide broad oversight of protein acylation during HSV infection, a roadmap for similar analysis in other systems, and a resource with which to pursue specific analysis of systems and functions.

INTRODUCTION

Covalent modification of proteins by saturated fatty acids represents a critical regulatory mechanism in processes including membrane and microdomain targeting, vesicular transport, signaling, cell structure, metabolic pathways, neurotransmission, infection, and immunity (Aicart-Ramos et al., 2011; Bijlmakers, 2009; Iwanaga et al., 2009; Resh, 2012). Examples in infection and immunity include LCK function, wherein mutants with amino acid substitutions that block myristoylation or palmitoylation are unable to reconstitute T-cell activation (Kabouridis et al., 1997; Yasuda et al., 2000; Yurchak and Sefton, 1995). The role of LCK acylation is not restricted to a membrane-binding function, since a variant that localizes to the plasma membrane through a transmembrane domain instead of palmitoylation is unable to efficiently reconstitute T-cell receptor signaling (Kabouridis et al., 1997). Interferon-induced transmembrane protein 3 (IFITM3) is involved in innate responses to flu, dengue fever, and West Nile virus (Brass et al., 2009), and it has recently been shown that *S*-palmitoylation of IFITM3 controls clustering in membrane compartments and its antiviral activity (Yount et al., 2010). Analysis of the family suggests that *S*-palmitoylation may be an ancient post-translational modification that is crucial for host resistance to viral infections. In addition to modulating compartmentalization, stability, and function of host proteins, lipid modification of virus-encoded proteins also plays a key role in virus replication and immune responses to infection. For example, in HIV, gp41 is palmitoylated at internal sites while Gag is subject to N-terminal myristoylation promoting incorporation of Gag into lipid rafts, multimerization, and particle budding (Resh, 2004). In hepatitis B virus myristoylation of surface protein sAg is required for infectivity (Gripon et al., 1995; Macrae et al., 1991). In this case a C-terminal transmembrane region anchors sAg protein to the virus envelope membrane and the myristoylated N-terminus on the virus exterior is involved in cell entry (Bruss et al., 1996). In the herpesviruses UL11, a conserved and essential structural protein, is subject to both myristoylation and palmitoylation, modifications that are essential for normal membrane association (Loomis et al., 2001; MacLean et al., 1989). Both human cytomegalovirus and Epstein-Barr virus encode homologs of UL11 (UL99 and BBLF1, respectively), which have also been shown to be lipidated; the precise nature of the lipidation in relation to virus assembly remains to be determined, but these proteins are all required for virus replication (Chiu et al., 2012; Sanchez et al., 2000). It has been also reported that another herpes simplex virus (HSV) tegument protein, UL51, undergoes palmitoylation, although the importance of this modification has not been studied in detail (Nozawa et al., 2003). From these and other examples, it is clear that covalent modification of host- and virus-encoded proteins plays important roles in many aspects of virus-host interaction including entry, assembly, and immune responses.

Studies on the role of protein acylation during infection are frequently reported on an individual case-by-case basis rather than from systematic unbiased analysis using high-throughput techniques such as mass spectrometry (MS)-based proteomic approaches. While advances in MS enable global profiling of many post-translational modifications, identification and quantification

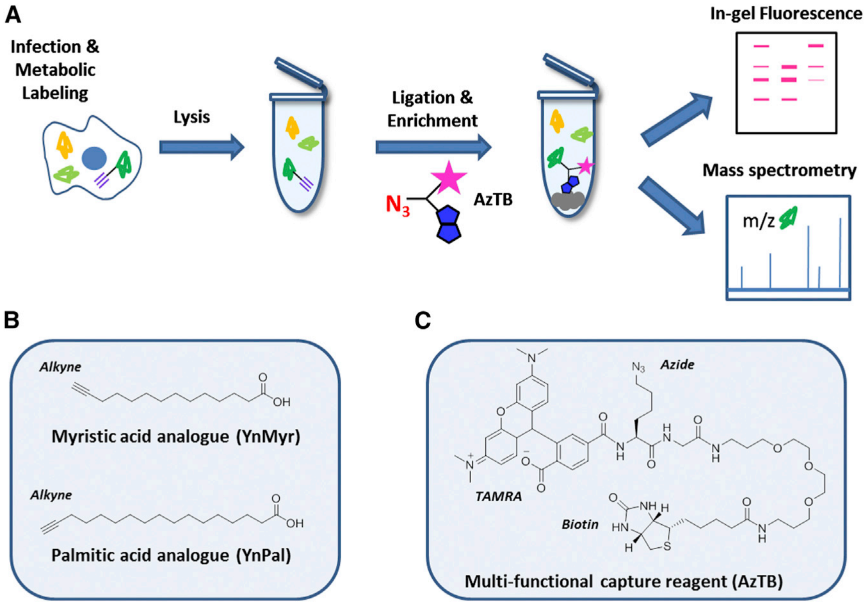

Figure 1. Enrichment Strategy

Chemoproteomic Workflow (A), Structures of Lipid Probes (B), and Multi-functional Capture Reagent AzTB (C). Inside the cell and the test tube, colored shapes represent proteins, the triple bar represents alkyne; on the capture reagent (AzTB), red N_3 represents azide, the pink star represents TAMRA fluorophore, and the blue double pentagon represents biotin.

of protein fatty acylation by standard MS remains very challenging. Acylation negatively affects solubility, chromatographic properties, and ionization of lipid-anchored tryptic peptides, rendering MS detection difficult. Furthermore, lipidated proteins often exist in low abundance in cells; therefore, lipid-specific enrichment is required to reduce sample complexity and improve discovery. An enrichment strategy that utilizes introduction of a chemical reporter via metabolic tagging with lipid probes in cell culture (Figure 1A) has become a method of choice to study protein myristoylation and palmitoylation, as well as other classes of lipidation (Berry et al., 2010; Broncel et al., 2015; Ciepla et al., 2014; Konitsiotis et al., 2015; Martin and Cravatt, 2009; Tate et al., 2015; Wilson et al., 2011; Wright et al., 2015). ω-Alkynyl fatty acid analogs (Figure 1B) can be used for metabolic tagging of acylated proteins and subsequently elaborated by a highly selective ligation reaction (copper-catalyzed azide alkyne cycloaddition [CuAAC]) (Rostovtsev et al., 2002; Tornoe et al., 2002) to a multi-functional azido capture reagent (AzTB, Figure 1C) enabling visualization (via the TAMRA component), enrichment (via the biotin component), and subsequent steps of on-bead tryptic digestion, MS measurement, software-aided peptide sequencing and identification, as well as quantification of fatty acylated proteins (Storck et al., 2013). In the work reported here, we applied chemical technologies with systems proteomic approaches to make comprehensive advances in the investigation of fatty acylation of virus and host proteins in a complex but tractable system with major clinical relevance in human disease, the human herpesviruses.

RESULTS

Selective Modulation of Protein Acylation During HSV Infection

In a series of preliminary experiments, uninfected cells were incubated with a range of concentrations (1–50 μM) of the probes for protein palmitoylation (YnPal) and myristoylation (YnMyr). The results indicated a concentration of 25 μM was optimal for both probes, with no apparent toxicity observed compared with vehicle (DMSO) over the 24-hr exposure time as measured by viable cell counting (Figure S1). Cells were then mock infected or infected with HSV-1[17] at an MOI of 5, i.e. an average of five infectious particles per cell, ensuring quantitative infection of all the cells for comparison with uninfected samples, and then pulse-labeled with YnPal and YnMyr lipid probes. Metabolically tagged, acylated proteins were subsequently ligated to AzTB, extracted, and visualized firstly by in-gel fluorescence (Figure 2). Distinct species were observed for HSV-infected versus mock-infected cells. For palmitoylation, candidate host species (Figure 2, open arrowheads) progressively reduced in abundance while a series of new species were observed, increasing with time after infection (Figure 2, solid arrows, lanes 1–4). A generally similar trend was observed for the myristoylation probe (Figure 2, solid arrows, lanes 5–8), with certain species migrating similarly to those observed with the palmitoylation probe.

These results demonstrate progressive alteration in the acylated protein profiles. Nevertheless, qualitative analysis by SDS-PAGE and in-gel fluorescence, while useful as a preliminary survey, is clearly limited for the ambition of a more broad-based analysis, including target protein identification. We therefore next employed MS combined with isotopic tagging methodology, stable isotope labeling by amino acid in cell culture (SILAC) (Ong et al., 2002), to identify and quantitate HSV-induced changes to lipid fatty acylation. In this approach (Figure 3A) cells to be infected are labeled with medium containing $^{13}C_6{}^{15}N_4$-arginine and $^{13}C_6{}^{15}N_2$-lysine (heavy), whereas cells to be mock infected are incubated in medium with arginine and lysine at natural isotopic distribution (light). Cells were infected or mock infected, incubated in heavy or light medium, respectively, and pulsed with the lipid probes. Mock-infected or infected cells were then harvested (19 hr post infection [hpi]) and the proteins extracted. After determining the total concentrations, proteins were mixed in a 1:1 ratio, subject to CuAAC ligation to AzTB, and affinity enriched on NeutrAvidin beads. The labeled, purified proteins were then subject to tryptic digestion and MS measurements (see Experimental Procedures). As part of this work flow, the isotope labeling was also reversed, whereby HSV-infected cells were incubated in light medium and mock-infected cells in heavy medium, thus minimizing experimental bias and aiding identification of incidental contaminants. For each probe, biological quadruplicate experiments were performed, measurement outcomes pooled, and proteins identified on a strict filtering basis requiring presence in replicate samples for inclusion in subsequent evaluation. The data were then further subjected to

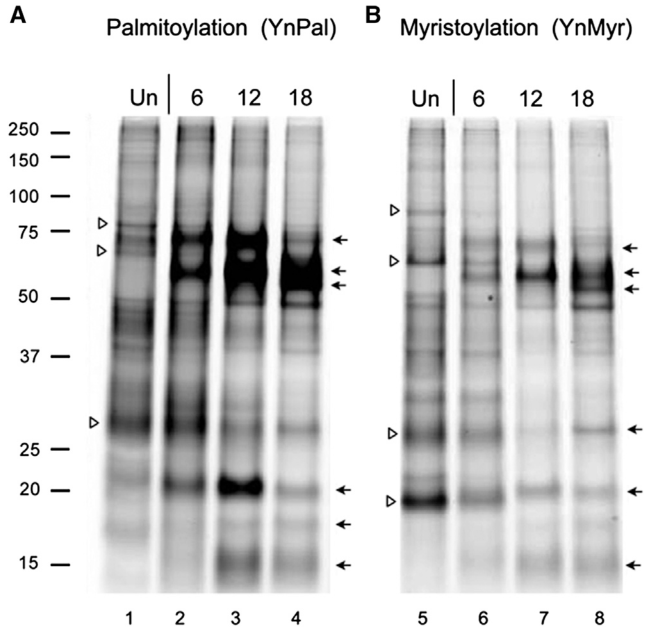

Figure 2. In-Gel Fluorescent Analysis of Protein Fatty Acylation During HSV Infection in RPE-1 Cells

Cells were pulsed with YnPal or YnMyr (25 μM) for 6 hr and harvested at various times as shown. Proteins were isolated, processed for coupling to capture reagent AzTB, and analyzed for palmitoylation (A) or myristoylation (B). Arrows point to bands of increased fluorescence intensities (proteins with increased acylation levels) and arrowheads point to bands with decreased fluorescence intensities (proteins with decreased acylation levels).

statistical analysis to identify proteins with significantly different lipidation status in infected and uninfected cells.

This analysis returned information on 1,292 grouped protein candidates for palmitoylation and 208 for myristoylation, revealing overall down-regulation of both protein modifications in HSV-infected cells compared with uninfected cells (Figures 3B and 3C, respectively). No host proteins were identified with increases in palmitoylation or myristoylation levels, attributing the increase in acylated species during infection (Figure 2) to HSV-encoded proteins.

Each dot in the Figures 3B and 3C represents an individual protein (or a small group of closely related proteins, e.g. isoforms) (Cox et al., 2011), and the complete datasets are summarized in Tables S1 and S2. Of 1,292 host protein groups enriched upon tagging with YnPal (Table S1), significant decreases in palmitoylation in HSV-infected cells were observed for 116 proteins, out of which 107 (92%) were annotated as intrinsic to a membrane. Of the 116 proteins (Table S1) with reduced levels of palmitoylation (>2-fold decrease) a number of proteins were quantified, including CXADR (Coxsackie virus and adenovirus receptor), ANO6, a protein involved in regulating membrane phospholipid asymmetry, and members of the tetraspanin family, e.g. CD63, CD81, and CD82, several of which are known to be palmitoylated and to form microdomains in the plasma membrane. Of the 208 proteins identified for myristoylation (Figure 3C), 107 exhibited reduced levels of myristoylation (>2-fold decrease, Table S2). Of these, 80 have been robustly validated as myristoylated proteins (Broncel et al., 2015; Thinon et al., 2014).

Selective Suppression of Host Protein Palmitoylation During HSV Infection

While these data catalog reduction in myristoylation and palmitoylation of host proteins during HSV infection, further analyses were nevertheless required to examine selectivity in these effects. This stems from the consideration that while myristoylation is generally a co-translational irreversible process linked to nascent synthesis of potential substrates, palmitoylation is a post-translational modification that is often dynamic and therefore affected by the total abundance of substrate proteins. In addition, palmitoylation of certain proteins occurs shortly after their biosynthesis and undergoes minimal turnover (Martin et al., 2012). As a result, certain aspects of acylation could be sensitive to host protein synthesis shutoff events induced by viruses such as HSV (Everly et al., 2002; Read and Frenkel, 1983; Suzutani et al., 2000).

We therefore next performed a series of additional analyses to take account of first, the total levels of protein substrates; second, the levels of nascent protein synthesis in infected versus uninfected cells; and third, the effect of protein synthesis inhibition per se. For the first two aims, we performed a set of SILAC-MS-based analyses in which total as well as newly synthesized protein abundances were quantified in HSV-infected and mock-infected cells. In the first analysis, mock-infected (light) and HSV-infected (heavy) samples from the workflow in Figure 3A were mixed in a 1:1 ratio, and the total sample prior to enrichment subject to tryptic digestion, MS, and quantitative analysis of the ratios of proteins in the total proteome was performed. The experiment revealed no significant alterations to abundances of the vast majority (99.6%) of 1,428 host protein groups that were rigorously quantified (Table S3). We noted that total abundances of a small number of proteins were significantly decreased in HSV-infected samples, including LRPPRC, PLD3, PRKDC, SERPINE1, and TGOLN2, none of which has been annotated as acylated. These data suggest that the observed down-regulation of acylation does not simply reflect differential total protein abundances.

In the next experiments, we examined alterations of nascent protein abundance in infected versus uninfected cells, and their relationship to protein acylation using a SILAC/pulse-chase protocol (Figure 4A). Cells in light medium were mock infected or infected and transferred to heavy medium, and incubation continued to compare heavy isotope incorporation into nascent proteins during the labeling period in uninfected versus HSV-infected cells. Equal amounts of the mock-infected and infected lysates were spiked with a fixed amount of protein from cells labeled by SILAC with an intermediate (medium) isotopic label, enabling normalization between samples through a standard "spike-in SILAC" approach (Geiger et al., 2011). Since we anticipated that these data might return only a small set particularly for the myristoylated proteins, we included a pulse with YnMyr prior to isotope labeling to enable enrichment and detection of this set, alongside the nascent analysis for all proteins. The spiked mixtures of total proteins were then subjected to tryptic digestion and MS analysis. In parallel, the YnMyr-tagged protein populations from the same lysate mixtures were ligated to AzTB, affinity-captured, and analyzed by on-bead digest and MS. The ratio of heavy/medium signal for mock- and HSV-infected samples yields information on changes in the abundance

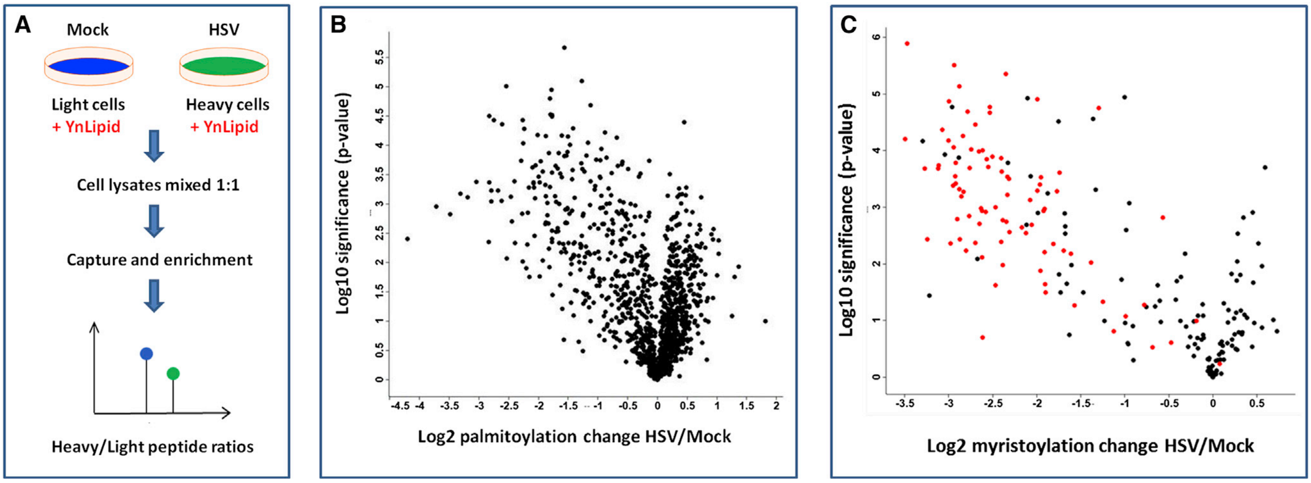

Figure 3. Quantitative Proteomics Analysis of Host Protein Fatty Acylation During HSV Infection in RPE-1 Cells

(A) SILAC-based quantitative proteomics workflow.

(B) Virus-induced changes to protein palmitoylation (n = 4) plotted against statistical significance of the ratio measured.

(C) Virus-induced changes to protein myristoylation (n = 4) plotted against statistical significance. Black, proteins with myristoylation requirement (N-terminal Gly); red, validated NMT substrates (Broncel et al., 2015; Thinon et al., 2014).

In (B) and (C) each data point represents a protein or a protein group (Cox et al., 2011).

of individual proteins (all proteins) synthesized during the time frame of the experiment, and the affinity-captured sample gives the same information specifically for the myristoylated population. The results for nascent protein abundance in HSV- versus mock-infected cells, plotted against significance of the difference for all proteins, are shown in Figure 4B. Overall the results for 510 proteins quantitated (Table S4) revealed a significant down-regulation of protein synthesis in HSV-infected cells, in keeping with previous data (Suzutani et al., 2000), consistent with the known progression of HSV infection. To selectively examine nascent abundances for myristoylated proteins, the average fold (Log_2) decrease among potential substrates (with myristoylation recognition motifs) was −1.86 ± 0.53 (33 protein groups). The average fold decrease among substrates purified by affinity enrichment for YnMyr and satisfying the criteria of possession of an N-terminal glycine and previous identification as myristoylated proteins (Broncel et al., 2015; Thinon et al., 2014) was −3.17 ± 0.23 (eight protein groups) (Table S4). Therefore, comparing the decrease in levels of nascent protein abundances from this analysis with the decrease in levels of myristoylation among validated substrates (Table S2), the effects were −2.33 ± 0.69 and −3.17 ± 0.23, respectively. These data suggest, therefore, that the apparent decrease in host protein myristoylation largely results from suppression of host protein synthesis in infected cells. In support of this, we note that total abundances of NMT1 and NMT2 in HSV- and mock-infected cells were very similar (Table S3), with average ratios of 1.13 ± 0.18 (four samples) and 1.12 ± 0.25 (seven samples), respectively.

In contrast, with regard to HSV effects on palmitoylation versus the effects on nascent protein abundances, similar cross-referencing of the data (383 proteins common to both datasets) revealed no correlation between the relative decreases in palmitoylation and the relative decreases in nascent protein abundances (data not shown; see also Figure S2). However, to further substantiate the proposal that the effect of HSV infection is not due simply to an effect on nascent protein synthesis, we performed further analysis to compare the effect of protein synthesis shutoff per se with that due to the complex events associated with HSV infection. If the effect of HSV infection on palmitoylation was related to an effect of protein synthesis inhibition per se, then we would expect a correlation between the datasets for HSV-suppressed palmitoylation and those whose palmitoylation is affected by protein synthesis inhibition. Thus relative levels of protein palmitoylation were quantified in uninfected cells in the absence and presence of cycloheximide (Chx), a well-established protein synthesis inhibitor. Using a SILAC protocol, cells in either light or heavy medium were incubated for 2 hr with YnPal. Chx (50 μg/ml) was then added to heavy cells but not to the light cells, and YnPal labeling continued in both sets of cultures for an additional 4 hr. Samples were then extracted, heavy and light proteins were mixed in a 1:1 ratio and ligated to AzTB, and palmitoylated proteins affinity enriched. Following MS measurements, quantification data were returned for 496 proteins. As expected, levels of palmitoylation were affected by Chx to different degrees for different proteins (Table S5). Cross-comparison of the datasets representing relative palmitoylation levels (for 495 proteins quantified in both experimental sets) as a function of HSV infection or Chx treatment revealed no clear correlation between responses to HSV and Chx (Figure S2; Table S6). Taken together, these data indicate that mechanisms other than host protein synthesis shutoff are involved in selective control of palmitoylation levels during HSV infection.

Identification of Novel Fatty Acylated HSV-Encoded Proteins

We next directed our attention to identification of putative viral proteins that were acylated by the host machinery during infection. The workflow depicted in Figure 3A was designed to

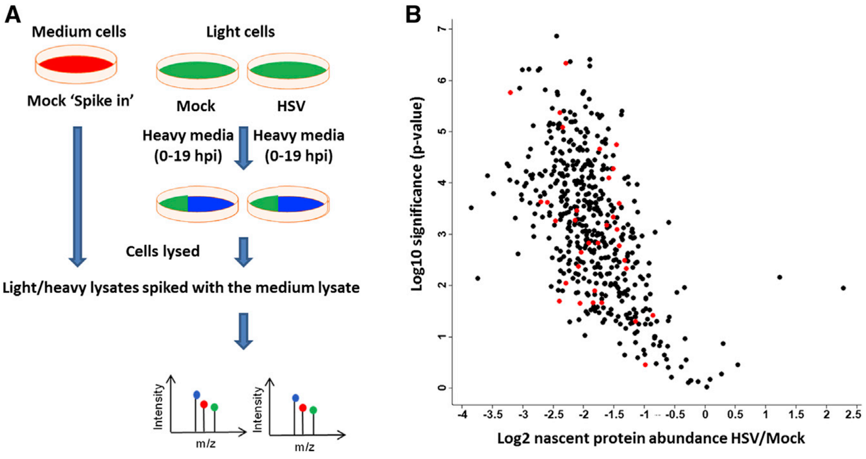

Figure 4. Infection-Induced Changes to Nascent Protein Abundances

(A) SILAC-based proteomic workflow.

(B) Experimental results from samples prior to affinity enrichment: Log2 nascent protein abundance ratio HSV/Mock (n = 3) plotted against significance of the change measured. Red, proteins with myristoylation requirement (N-terminal Gly); black, all other proteins quantified. Each data point represents a protein or a protein group (Cox et al., 2011).

measure HSV-induced changes to the host proteome and was not optimal for an unambiguous identification of fatty acylated viral proteins. Therefore to discriminate between bona fide fatty acylated proteins and abundant HSV-encoded proteins which might non-specifically bind to the capture beads, we modified our quantitative metabolic tagging workflow (Figure 5A). Thus, heavy and light cells were infected (n = 3) with HSV and incubated with either the alkynyl fatty acid probes (heavy) or the natural fatty acids (light). The natural precursor does not contain a bio-orthogonal tag (alkyne moiety) and does not undergo chemoselective ligation to AzTB, thus significant heavy/light signal enrichment would be expected for fatty acylated proteins over non-specifically binding proteins. Using parallel pairs of probes, YnPal and Pal (palmitic acid) or YnMyr and Myr (myristic acid), nine putative palmitoylated and five putative myristoylated HSV proteins were identified in two or more samples (Figure S3; Table S7). The selective and highly significant identification of glycoproteins among the HSV proteome suggests that the assignments are highly robust. The set includes gE, gI, gG, gK, US2, US3, UL56, UL51 (TEG7), and UL24, and, consistent with a lack of myristoylation consensus in these proteins except for US2, proteins identified with YnMyr may in fact incorporate this probe through the palmitoylation pathway. Results supporting this conclusion were shown from the observed lack of sensitivity of YnMyr incorporation into HSV-encoded proteins to a specific and potent myristoylation inhibitor (Frearson et al., 2010; Goncalves et al., 2012; Table S8). In this analysis we quantified changes in the YnMyr incorporation for 9 out of 11 HSV proteins possessing a myristoylation motif. UL11 (a known myristoylated and palmitoylated protein) (Loomis et al., 2001; MacLean et al., 1989) and gN were not detected in our analyses. A reasonable explanation for this is the very limited number of tryptic peptides in these small proteins that are compatible with MS and tandem MS (MS/MS) detection.

To support our analysis, we examined palmitoylation in two other herpesvirus strains, HSV-1[KOS] and HSV-2[186] as summarized in Figure 5B and Table S7. Of 12 putatively palmitoylated HSV proteins which constitute over 15% of the viral proteome (Apweiler et al., 2004), seven (gE, gI, gK, UL51, UL24, US2, US3) were common for all three strains and one (UL56) for at least two strains, whereas one (gH) was shown to be specific for HSV-2 and one (gG) to be specific for HSV-1.

In additional work, we employed acyl-RAC (resin-assisted capture), an orthogonal approach to profile *S*-fatty acylation of proteins, to support the identification of HSV-encoded protein palmitoylation and provide additional information on the sites of *S*-acylation in these proteins (Forrester et al., 2011). In brief, free cysteines on proteins were capped, thioesters selectively hydrolyzed with hydroxylamine (this step is performed without hydroxylamine in the control sample), newly exposed free cysteines captured with a thiol-reactive resin, and bulk protein removed by on-resin digestion. Finally, captured peptides were selectively released from the resin and sequenced by MS/MS, permitting indirect identification of the cysteine on which *S*-acylation was originally installed. Thus cells were infected (n = 3) with HSV-1[17] and protein lysates subjected to acyl-RAC, revealing eight distinct *S*-fatty acylation sites on five of the HSV proteins (Table 1) that were also identified as palmitoylated by the method described above.

DISCUSSION

We report a pathway and workflow to enable whole-proteome insights into protein acylation during infection with a large complex DNA virus, HSV. Our work highlights the coupling of dramatic reductions in protein myristoylation to the suppression of nascent protein synthesis and the identification of several specific host palmitoylated proteins whose modification is specifically suppressed during infection. The annotated functions of these latter proteins relate predominantly to membrane compartmentalization and protein trafficking, likely reflecting selective modulation of these pathways by altered palmitoylation during the progression of infection. In this regard it is noteworthy that protein *S*-palmitoylation is largely mediated by the zDHHC family of membrane-associated enzymes, which may be themselves regulated by palmitoylation. Our dataset includes relative palmitoylation levels for 11 zDHHC enzymes of which selective underpalmitoylation in infected cells was observed for zDHHC20, a zDHHC member that exhibits substrate specificity for a number of integral membrane proteins (Ohno et al., 2012). Down-regulation of zDHHC20 could contribute to the observed protein underpalmitoylation of integral membrane proteins reported above, although clearly other mechanisms, including suppressed activities of *S*-palmitoyl transferases that were not returned in our dataset or enhanced activities of acyl protein thioesterases, may be involved. Of the target set with selectively reduced palmitoylation, gene

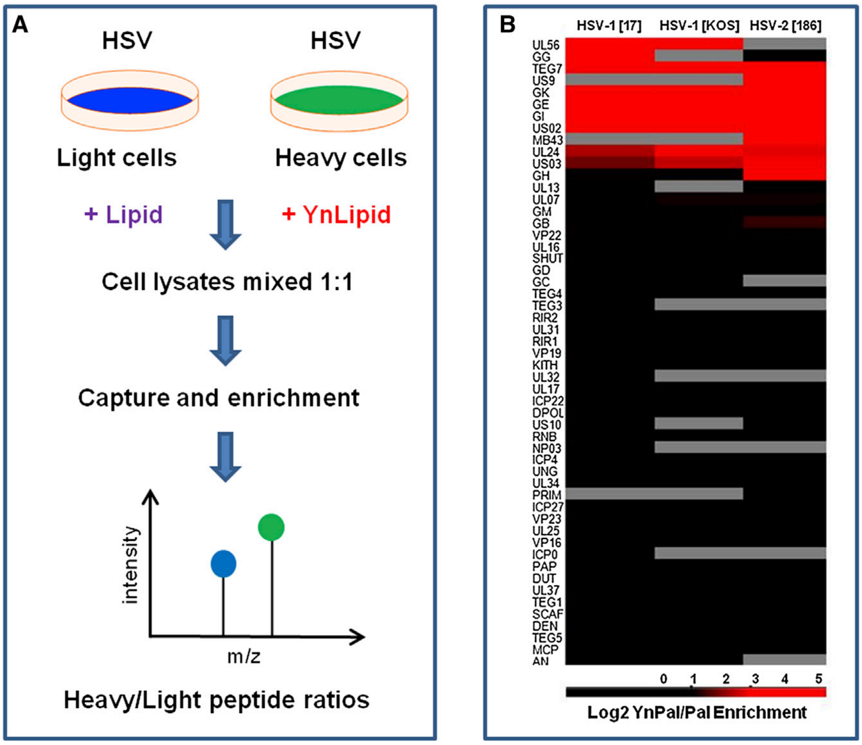

Figure 5. SILAC-Based Identification of Fatty Acylated HSV-Encoded Proteins

(A) SILAC-based experimental workflow.

(B) Heatmap for YnPal/Pal (25 μM, 5–19 hpi) enrichment (n = 3) of proteins derived from HSV-1 [17], HSV-1[KOS], and HSV-2[186]. Black to red gradation represents the degree (n = 3) of enrichment of proteins labeled with YnPal over Pal, expressed as a heatmap. Gray bars represent missing values (applied when quantification was missing for more than one out of three samples).

ontology annotation analysis (Table S9) revealed statistically significant enrichment in terms (Huang et al., 2009) including location to plasma membrane (63 proteins) and Golgi apparatus (26 proteins), and involvement in processes such as vesicle-mediated transport (19 proteins) and ion transport (17 proteins). As indicated above, the most commonly enriched protein domain term was tetraspanin (9 proteins), and the most populated pathway was SNARE interactions in vesicular transport (8 proteins).

We further provide robust data expanding the list of HSV-encoded proteins subject to acylation, and demonstrate that cysteine *S*-acylation predominates, with differences in modification in HSV-1 versus HSV-2 for at least two modified proteins. Previous work has demonstrated that numerous viruses encode palmitoylated proteins that play important roles in the process of virus assembly and release, and, as discussed in the introduction, herpesvirus-encoded palmitoylated proteins have been identified. Here we expand significantly on the number of HSV-encoded acylated proteins and identify in particular gE, gI, gK, and the proteins US9, US2, and US3, each of which play multiple and key roles during infection, including viral entry and release, cell-to-cell spread, viral immune evasion, and inhibition of apoptosis. Considering in particular that palmitoylation modification is reversible, subject to cycles of palmitoylation/depalmitoylation it can be involved in the regulation of diverse aspects of subcellular localization, conformation, protein-protein interactions, and activity of proteins. Our work now sets the stage to pursue functional analysis of the role of acylation in these proteins using a combination of approaches including, for example, the effect of inhibitors on activity and localization and site-directed mutagenesis of the individual residues subject to modification.

SIGNIFICANCE

This study represents the first example of modern chemical proteomics tools, namely bio-orthogonal probes and capture reagents, applied systematically to investigate protein fatty acylation events on both host- and virus-encoded proteins during viral infection. With this technology, we were able to quantify infection-induced changes to fatty acylation of hundreds of host proteins (some of which are involved in immune response and modulation of the progression of infection). Furthermore, we also demonstrate increased sensitivity of the technique over previous routes, and identify novel virus-encoded proteins, indicating that a significant fraction of viral proteome is subject to fatty acylation. Having established a robust workflow for thorough analysis of acylation during HSV infection, our work sets the stage for further studies aiming to decipher the importance of fatty acylation to host and viral protein function and location in cells, and in the formation of viral particles.

EXPERIMENTAL PROCEDURES

Chemical Tools

Tetradec-13-ynoic acid (YnMyr) (Goncalves et al., 2012), heptadec-16-ynoic acid (YnPal) (Milne et al., 2010), *N*-myristoyl transferase inhibitor (DDD85646) (Frearson et al., 2010; Goncalves et al., 2012), and AzTB (Heal et al., 2012) were synthesized as described previously. Myristic (Myr) and palmitic (Pal) acids and all other chemicals were purchased.

Cells and Viruses

RPE-1 (immortalized retinal pigment epithelial) cells were grown in DMEM-F12 medium containing 10% serum, 100 mM L-glutamine, and penicillin-streptomycin, in a humidified 5% CO_2 containing atmosphere at 37°C. Cells were plated at least 24 hr before treatment with chemical probes and/or virus infection. Virus strains have been previously described and included HSV-1[17], HSV-1[KOS], and HSV-2[186].

Metabolic Tagging Experiments with In-Gel Fluorescent Detection

Cells were inoculated with serum-free DMEM-F12 media in the presence (infection) or absence (mock) of HSV at MOI = 5 for 1 hr. The media were removed and replaced by fresh DMEM-F12 containing 2% newborn calf serum (NCS) and lipid probes (25 μM YnMyr or YnPal). The cells were washed with ice-cold PBS (2×) and harvested at 19 hpi in SDS lysis buffer. For time-course experiments, the cells were pulse incubated with the probes at

Table 1. Palmitoylation Sites on Proteins Identified by Acyl-RAC

Gene	Modified Sequence	Site	Sample	z	m/z	Error[a]	Score	Δ Score	Intensity
gG	_YVCLPSERG	C232	A	2	540.7608	0.2964	105.1	96.866	27,290,000
gG	_YVCLPSERG	C232	A	2	540.7608	0.7457	53.76	48.216	3,025,400
gG	_YVCLPSERG	C232	A	2	540.7608	0.4422	53.76	48.216	3,450,800
gG	_YVCLPSERG	C232	B	2	540.7608	0.0884	117.9	100.38	68,265,000
gG	_YVCLPSERG	C232	B	2	540.7608	0.2183	74.46	67.185	7,849,900
gG	_YVCLPSERG	C232	C	2	540.7608	0.5153	108.7	100.16	9,156,000
gI	_RPAVAFALCR_	C108	B	3	387.5501	−0.2648	61.77	52.059	8,923,800
gI	_RPAVAFALCR_	C108	B	3	387.5501	−0.1724	69.28	59.495	8,617,300
gI	_RPAVAFALCR_	C108	B	3	387.5501	−0.2043	58.7	52.386	2,518,600
gI	_RPAVAFALCR_	C108	C	3	387.5501	0.5562	60.4	50.743	910,210
gI	_RPIYSPQMPTGISCAVNEAAMAR_	C321	B	3	840.7434	−0.1068	37.37	34.878	2,856,600
gI	_RPIYSPQMPTGISCAVNEAAMAR_	C321	C	3	840.7434	−0.5254	37.82	35.717	3,090,200
TEG7	_AAPCVLGQ	C240	A	2	408.2076	−0.3092	35.36	31.964	11,740,000
TEG7	_AAPCVLGQ	C240	B	2	408.2076	−0.2111	46.02	42.624	21,662,000
TEG7	_TYHACMVNLER_	C78	A	3	465.2166	−0.2680	52.39	43.981	8,275,000
TEG7	_TYHACMVNLER_	C78	B	3	465.2166	−0.0848	37.14	30.708	5,543,200
TEG7	_TYHACMVNLER_	C78	B	3	465.2166	0.2983	60.64	36.918	6,623,500
TEG7	_TYHACMVNLER_	C78	B	3	465.2166	−0.0930	88.13	74.325	23,976,000
TEG7	_TYHACMVNLER_	C78	B	3	465.2166	−0.0122	67.93	50.191	17,289,000
TEG7	_TYHACMVNLER_	C78	B	3	465.2166	0.2979	43.9	35.487	8,906,500
UL24	_IAALFCVPVATK_	C255	A	2	645.3679	−0.1287	85.55	72.154	15,173,000
UL24	_IAALFCVPVATK_	C255	B	2	645.3679	−0.0379	96.82	83.42	20,055,000
UL24	_IAALFCVPVATK_	C255	C	2	645.3679	0.0554	95.42	82.017	14,522,000
US02	_LPHICYPVTTL	C285	A	2	657.3497	−0.0473	62.56	53.857	5,930,000
US02	_LPHICYPVTTL	C286	B	2	657.3497	0.1274	64.71	59.496	5,752,800
US02	_LPHICYPVTTL	C287	C	2	657.3497	0.0611	59.15	53.937	4,252,600
US02	_RPCCACMGMPEVPDEQPTSPGR_	C244[b]	A	3	844.6901	−0.4280	51.88	51.761	17,659,000
US02	_RPCCACMGMPEVPDEQPTSPGR_	C244[b]	B	3	844.6901	−0.3033	34.65	34.404	8,687,600
US02	_RPCCACMGMPEVPDEQPTSPGR_	C244[b]	C	3	844.6901	−0.7995	42.35	42.348	4,100,600

[a]Error given in ppm.
[b]Alternative sites exist on this peptide (C245 and C247), and cannot be distinguished by Acyl-RAC.

1–7 hpi, 7–13 hpi, and 13–19 hpi, and harvested at the end of the respective pulse.

Metabolic Tagging Experiments with MS Detection and SILAC-Based Quantification

Cells referred to as heavy were cultured in SILAC R10K8 DMEM-F12 medium containing $^{13}C_6{}^{15}N_4$-Arginine and $^{13}C_6{}^{15}N_2$-lysine supplemented with 10% dialyzed fetal bovine serum (FBS) for at least five passages (at least ten doublings). Cell dissociation buffer was used to detach cells during splitting. Cells referred to as intermediate and light were treated analogously, but the SILAC R6K4 DMEM-F12 medium they were grown in contained $^{13}C_6$-arginine and $^{2}H_4$-lysine or arginine and lysine at their natural isotopic abundances, respectively.

Differential Levels of Lipidation in Virus- and Mock-Infected Cells

Four populations of heavy cells and four populations of light cells (2×10^7 cells each) were infected with HSV-1[17] at MOI = 5 or mock infected in serum-free SILAC DMEM-F12 media for 1 hr. Media were removed and replaced by fresh SILAC DMEM-F12 supplemented with 2% dialyzed FBS and lipid probes (25 μM YnMyr or YnPal). At 19 hpi, the cells were washed with ice-cold PBS (2×) and harvested in SDS lysis buffer (500 μl). Samples were probe sonicated on ice and spun at 5,000 × *g* for 20 min to remove insoluble nuclear material. Protein concentration was determined and the samples were stored at −80°C awaiting further processing. Prior to the next processing step, heavy virus-infected lysates were mixed with light mock-infected lysates and light virus-infected lysates were mixed with heavy mock-infected lysates, in 1:1 ratio (based on the protein concentration determined). Sample mixtures were either digested in-solution or on-bead (following chemical ligation and affinity enrichment).

Determination of Lipidated HSV-Encoded Proteins

For each virus applied, three populations of heavy cells and three populations of light cells (2×10^7 cells each) were infected at MOI = 5 in serum-free SILAC DMEM-F12 media for 1 hr. Media were removed and replaced by fresh SILAC DMEM-F12 supplemented with 2% dialyzed FBS. At 6 hpi, heavy media were supplemented with lipid probes (40 μM YnMyr or YnPal) and light media were supplemented with natural lipids (40 μM Myr or Pal). At 19 hpi, the cells were washed with ice-cold PBS (2×) and harvested in SDS lysis buffer (500 μl).

Differential Levels of Lipidation in Cells Cultured in the Presence or Absence of Cycloheximide

In experiments involving protein synthesis inhibitor (Chx), two populations of light cells and two populations of heavy cells (n = 2) were incubated with YnPal (25 μM) for 2 hr prior to the addition of Chx (50 mg/ml) to the heavy cells, and incubation continued for another 4 hr. Cells were washed with ice-cold PBS (2×) and harvested in SDS lysis buffer (500 μl). Samples were probe sonicated on ice and spun at 5,000 × *g* for 20 min to remove insoluble nuclear material. Protein concentration was determined and the samples were stored at −80°C to await further processing. Prior to the next processing step (chemical

ligation), heavy lysates were mixed with light lysates in 1:1 ratio (based on the protein concentration determined).

In experiments involving *N*-myristoyltransferase inhibitor DDD85646, light cells were supplemented either with DDD85646 (5 μM) or the vehicle (DMSO) at 1 hpi together with YnMyr (25 μM). At 19 hpi, the cells were washed with ice-cold PBS (2×) and harvested in SDS lysis buffer (500 μl). "Spike-in standards" were generated by incubation of heavy cells with YnMyr (25 μM) from 1 to 19 hpi. Samples were probe sonicated on ice and spun at 5,000 × *g* for 20 min to remove insoluble nuclear material. Protein concentration was determined and the samples were stored at −80°C to await further processing. Prior to the next processing step (chemical ligation), heavy lysates were mixed with light lysates in 1:3 ratio (based on the protein concentration determined).

Differential Protein Synthesis Rates in Virus- and Mock-Infected Cells

Three populations of light cells (2 × 10^7 cells each) were infected with HSV-1 [17] at MOI = 5, and three populations of light cells (2 × 10^7 cells each) were mock infected in serum-free heavy SILAC R10K8 DMEM-F12 supplemented with YnMyr (25 μM) for 6 hr. Media were removed and replaced by fresh heavy SILAC 10K8 DMEM-F12 supplemented with 2% dialyzed FBS and YnMyr (25 μM). At 19 hpi, the cells were washed with ice-cold PBS (2×) and harvested in SDS lysis buffer. Samples were probe sonicated on ice and spun at 5,000 × *g* for 20 min to remove insoluble nuclear material. An additional population of cells cultured in intermediate SILAC R6K4 DMEM-F12 (uninfected) and metabolically tagged with YnMyr (25 μM, 8 hr) was harvested in SDS lysis buffer (and treated further as above) and applied as a spike-in standard (Geiger et al., 2011). Protein concentration was determined and the samples were stored at −80°C to await further processing. Before further processing, light-heavy and intermediate lysates were mixed at 4:1 ratio (based on the protein concentration determined). Sample mixtures were either digested in-solution or on-bead (following chemical ligation and affinity enrichment).

Chemical Ligation with CuAAC

Lysates were thawed on ice. 200 μg (in-gel fluorescence) or 800 μg (MS) of proteins were taken and diluted to 2 mg/ml using SDS lysis buffer and 1× PBS (final SDS concentration in samples was adjusted to 1%). A click mixture was prepared by adding reagents together in the following order and by vortex mixing between the addition of each reagent: AzTB (1 or 4 μl, stock solution 10 mM in water, final concentration 0.1 mM), $CuSO_4$ (2 or 8 μl, stock solution 50 mM in water, final concentration 1 mM), tris(2-carboxyethyl)phosphine (TCEP) (2 or 8 μl, stock solution 50 mM in water, final concentration 1 mM), tris(benzyltriazoylmethyl)amine (1 or 4 μl, stock solution 10 mM in DMSO, final concentration 0.1 mM). Following the addition of the click mixture (6 or 24 μl/sample) the samples were vortex mixed for 1 hr at room temperature (RT), and the reaction was stopped by addition of EDTA (2 or 8 μl, stock solution 500 mM in water, final concentration 10 mM). Subsequently, proteins were precipitated (chloroform/methanol/sample, 0.25:1:1 v/v/v), precipitated protein pellets isolated by centrifugation (17,000 × *g*, 10 min), washed twice with methanol (1,000 μl), and air dried. The pellets were then reconstituted in SDS lysis buffer, diluted to 0.4% SDS with PBS, and final protein concentration of 1 mg/ml. Insoluble material was removed by centrifugation.

Protein Enrichment on Avidin Beads

For proteomics experiments (400 μg of proteins), agarose beads (40 μl of pre-washed) were added to proteins in 0.4% SDS in PBS (1 mg/ml). The mixtures were gently vortex mixed for 90 min at RT. The supernatant was removed and the beads washed with 1% SDS in PBS (3 × 1 ml), and then with 100 mM ammonium bicarbonate (AMBIC) (3 × 1 ml). The beads were incubated with 5 mM TCEP in 100 mM AMBIC (100 μl) for 20 min at 55°C, washed with 50 mM AMBIC, and incubated with 5 mM iodoacetamide in 100 mM AMBIC (100 μl) for 20 min at RT in the dark. The beads were subsequently washed with 50 mM AMBIC (4 × 1 ml). On-bead tryptic digest was performed in the next step (see below).

For small-scale experiments (100 μg of proteins), magnetic beads (15 μl of pre-washed) were added to proteins in 0.4% SDS in PBS (1 mg/ml). The mixtures were gently vortex mixed for 90 min at RT. The supernatant was removed and the beads washed with 1% SDS in PBS (3 × 500 μl). Proteins were released from the beads in the next step (see below).

Determination of Palmitoylation Sites for HSV-Encoded Proteins

Three populations of cells (4 × 10^7 cells each) cultured in DMEM-F12 (non-SILAC) were infected with HSV-1[17] at MOI = 5 in serum-free DMEM-F12 for 1 hr. The media were removed and replaced by fresh DMEM-F12 supplemented with 2% NCS. At 19 hpi, the cells were washed with ice-cold PBS (2×) and harvested in 2% SDS in Tris buffer (100 mM, pH 7.5). Samples were probe sonicated on ice and spun at 5,000 × *g* for 20 min to remove insoluble nuclear material. Protein concentration was determined. The lysates (4 mg of proteins each) were diluted with Tris buffer (100 mM, pH 7.5) to the final SDS concentration of 1% and protein concentration of 2 mg/ml. To block non-palmitoylated cysteines, samples were treated with TCEP (final concentration 5 μM) and *N*-ethylmaleimide (final concentration 10 μM) for 30 min at 40°C. Proteins were precipitated (chloroform/methanol/sample 0.25:1:1 v/v/v), protein pellets isolated by centrifugation (17,000 × *g*, 10 min), washed with methanol (5 × 1 ml), and air dried. Proteins were reconstituted in 2% SDS in Tris buffer (100 mM, pH 7.5), diluted with Tris buffer (100 mM, pH 7.5) to the final SDS concentration of 1.5% and protein concentration of 3 mg/ml, and spun (17,000 × *g*, 10 min) to remove traces of insoluble material. Each sample was then divided into two parts, to produce two groups of three samples. One group was treated with freshly prepared hydroxylamine (500 μl of 1 M, stock solution prepared in 100 mM Tris buffer and pH adjusted to 7.5, final concentration 0.25 M), whereas the second (control) group was treated with NaCl (500 μl of 1 M, stock solution prepared in 100 mM Tris buffer and pH confirmed to be 7.5, final concentration 0.25 M) for 2 hr at RT, both in the presence of Thiopropyl Sepharose 6B resin (20 mg per sample, pre-swollen according to the manufacturer's recommendation). Hydroxylamine solution was produced by neutralization of hydroxylamine hydrochloride with NaOH, hence the addition of NaCl to the control samples. The resin was spun (7,000 × *g*, 4 min), supernatant discarded, and the resin washed with 2% SDS in Tris buffer (3 × 1 ml) and Tris buffer (3 × 1 ml). On-bead digestion was performed in the next step (see below).

SDS-PAGE and In-Gel Fluorescence

Total cell lysates were boiled in 1× SLB (NuPAGE LDS sample buffer; Invitrogen) for 5 min, whereas enriched proteins bound to magnetic beads were boiled in 1× SLB for 10 min. Supernatants separated from beads or cell samples loaded on Tris-acetate SDS-PAGE gels (12%). Following electrophoresis (90 min, 150 V), gels were soaked in a fixing solution (40% MeOH, 10% acetic acid, 50% water) and washed with water (3×), in-gel fluorescence was recorded, and the protein loading was analyzed by Coomassie staining.

Tryptic Digest and MS Sample Preparation

Proteins bound to NeutrAvidin-agarose beads (40 μl) were incubated with sequencing-grade trypsin (0.5 μg) in 50 mM AMBIC (100 μl) for 16–20 hr in a thermo-shaker set to 37°C. The reaction was terminated with trifluoroacetic acid (0.5 μl) and the beads spun (7,000 × *g*, 4 min). Supernatant was removed and kept aside. The beads were washed with 0.5% trifluoroacetic acid (50 μl), spun again (7,000 × *g*, 4 min) and the second supernatant combined with the one that was set aside. Tryptic peptides in the supernatant were desalted by the Stage-Tip method as described elsewhere (Broncel et al., 2015). Elution from the sorbent (SDB-XC poly(styrenedivinylbenzene) copolymer, from 3 M) with 70% acetonitrile in water was followed by Speed-Vac-assisted solvent removal. Peptides were reconstituted in water containing 0.5% trifluoroacetic acid and 2% acetonitrile and transfer of samples into liquid chromatography-MS sample vials.

Proteins bound to Thiopropyl Sepharose 6B resin were suspended in 100 mM Tris buffer (pH 8) (100 μl), sequencing-grade trypsin (0.5 μg) added, and the mixtures incubated for 16 hr in a thermo-shaker set to 37°C. The resin was spun (7,000 × *g*, 4 min), the supernatant discarded, resin washed with 2% SDS in Tris buffer (3 × 1 ml), Tris buffer (3 × 1 ml), 80% aqueous acetonitrile (3 × 1 ml), 50% aqueous methanol (3 × 1 ml), and 50 mM AMBIC (3 × 1 ml). Thiol-containing peptides were eluted from the resin by the incubation with DTT (final concentration 50 mM) for 40 min at 40°C. The resin was spun (7,000 × *g*, 4 min), supernatant withdrawn, desalted by the Stage-Tip method, and processed further as described above.

In-solution digestion was performed on non-enriched samples (total cell lysates). To remove the detergent, proteins (50 μg) were precipitated from lysis buffer solutions by addition of methanol (400 μl), chloroform (100 μl), and water (350 μl). Protein pellets were isolated by centrifugation (17,000 × *g*, 10 min),

washed (3×) with methanol (1,000 μl), and air dried. The pellets were then reconstituted in 50 mM AMBIC (100 μl), TCEP (5 μl, 100 mM) added, and the mixtures incubated for 20 min at 55°C. Subsequently, iodoacetamide (5 μl, 100 mM) was added and the mixtures left for 20 min at RT in the dark. Sequencing-grade trypsin (0.5 μg) was added and the mixtures were incubated for 16–20 hr in a thermo-shaker set to 37°C. The reaction was terminated with trifluoroacetic acid (0.5 μl), and the peptide mixtures were desalted by the Stage-Tip method and processed further as above.

Mass Spectrometry

The analysis was performed using an Acclaim PepMap RSLC column of 50 cm × 75 μm inner diameter (Thermo Fisher Scientific) using a 2-hr acetonitrile gradient in 0.1% aqueous formic acid at a flow rate of 250 nl/min. Easy nLC-1000 was coupled to a Q Exactive mass spectrometer via an easy-spray source (all Thermo Fisher Scientific). The Q Exactive was operated in data-dependent mode with survey scans acquired at a resolution of 75,000 at *m/z* 200 (transient time 256 ms). Up to ten of the most abundant isotope patterns with charge +2 or higher from the survey scan were selected with an isolation window of 3.0 *m/z* and fragmented by higher-energy collisional dissociation with normalized collision energies of 25. The maximum ion injection times for the survey scan and the MS/MS scans (acquired with a resolution of 17,500 at *m/z* 200) were 20 and 120 ms, respectively. The ion target value for MS was set to 10^6 and for MS/MS to 10^5, and the intensity threshold was set to 8.3×10^2.

Proteomics Data Analysis

The data were processed with MaxQuant (Cox and Mann, 2008) (versions 1.3 and 1.5) and the peptides were identified from the MS/MS spectra searched against reference human and either herpesvirus type 1 (strain 17) or herpesvirus type 2 (strain HG52), depending on the virus type used in the experiment, proteomes (Apweiler et al., 2004) using the Andromeda (Cox et al., 2011) search engine. Cysteine carbamidomethylation was selected as a fixed modification (except for acyl-RAC samples) and methionine oxidation as a variable modification. For in silico digests of reference proteomes the following peptide bond cleavages were allowed: arginine or lysine followed by any amino acid (a general setting referred to as trypsin/P). Heavy/intermediate/light isotopic forms of arginine and lysine were used where appropriate. Complete datasets that corresponded to a particular experiment were always analyzed together with match between runs, with the re-quantify function enabled in the software. Up to two missed cleavages were allowed. The false discovery rate was set to 0.01 for peptides, proteins, and sites. Other parameters were used as pre-set in the software. "Unique and razor peptides" mode was selected to allow identification and quantification of proteins in groups (razor peptides are uniquely assigned to protein groups and not to individual proteins). Data were further processed using Perseus software (version 1.5).

In the case of SILAC experiments, contaminants, reverse, and identified by site hits were removed, and SILAC ratio values were logarithmized (Log2). The replicates were grouped together, and at least two valid values across three (viral protein lipidation or host nascent protein abundance) or four (host protein lipidation) replicates were required. For each double SILAC experiment, a one-sample test (Benjamini-Hochberg false discovery rate = 0.05) implemented in the software was performed to assess statistical significance of differences between measured SILAC ratios and the median SILAC distribution for all quantified proteins (set to Log2 = 0). For the pulse-SILAC experiment (host nascent protein abundance), a two-sample test (permutation-based false discovery rate = 0.05) implemented in the software was performed to assess statistical significance of differences between SILAC ratios measured for HSV-infected and mock-infected samples. Palmitoylation data were used without filtering, whereas myristoylation data were filtered in Perseus through a modified fasta file comprising only N-terminal MG proteins (the sequence requirement for co-translational myristoylation).

SILAC samples prepared from YnPal labeling experiments in the presence or absence of Chx (two biological duplicates) were measured twice (technical duplicates), and the duplicate data pulled in during MaxQuant analysis to enhance identification and provide more robust quantification.

In the case of acyl-RAC, sequential filtering was performed in Perseus to minimize false discovery. Peptides belonging to the following categories were removed: contaminant and reverse; also identified in sodium chloride-treated (hydroxylamine untreated) control samples; corresponding to proteins neither quantified nor significantly enriched in YnPal/Pal experiments for HSV-1[17]; possessing delta score <30; found in less than two out of three samples.

The heatmap and two-dimensional charts (Figure 5B; Figures S2 and S3) were prepared in Perseus. Tables S1, S2, S3, S4, S5, S6, S7, and S8 were created in Excel from data exported as .txt files from Perseus. Functional annotation analysis (Table S9) was performed using David Bioinformatics Resources. Term enrichment analysis was performed with human proteome applied as a background and the following threshold: minimal count = 3; ease = 0.001.

SUPPLEMENTAL INFORMATION

Supplemental Information includes three figures and nine tables and can be found with this article online at http://dx.doi.org/10.1016/j.chembiol.2015.06.024.

AUTHOR CONTRIBUTIONS

R.A.S. performed sample handling and analysis downstream of cell culture. R.A.S. and F.A. performed metabolic tagging in cell culture and viral infections. R.A.S. and E.K. performed MS analyses, and R.A.S. performed proteomics data processing and analysis. P.O'H., E.W.T., and R.A.S. conceived the study and wrote the manuscript with input from all the other authors.

ACKNOWLEDGMENTS

This work was supported by the Medical Research Council (award number MR/L000148/1). The authors thank Lisa Haigh of the Department of Chemistry at Imperial College for assistance with the mass spectrometer, and the Tate lab members for synthesis of YnMyr, YnPal, and AzTB.

Received: April 21, 2015
Revised: June 19, 2015
Accepted: June 30, 2015
Published: August 6, 2015

REFERENCES

Aicart-Ramos, C., Valero, R.A., and Rodriguez-Crespo, I. (2011). Protein palmitoylation and subcellular trafficking. Biochim. Biophys. Acta *1808*, 2981–2994.

Apweiler, R., Bairoch, A., Wu, C.H., Barker, W.C., Boeckmann, B., Ferro, S., Gasteiger, E., Huang, H.Z., Lopez, R., Magrane, M., et al. (2004). UniProt: the Universal Protein knowledgebase. Nucleic Acids Res. *32*, D115–D119.

Berry, A.F.H., Heal, W.P., Tarafder, A.K., Tolmachova, T., Baron, R.A., Seabra, M.C., and Tate, E.W. (2010). Rapid multilabel detection of geranylgeranylated proteins by using bioorthogonal ligation chemistry. Chembiochem *11*, 771–773.

Bijlmakers, M.J. (2009). Protein acylation and localization in T cell signaling (Review). Mol. Membr. Biol. *26*, 93–103.

Brass, A.L., Huang, I.C., Benita, Y., John, S.P., Krishnan, M.N., Feeley, E.M., Ryan, B.J., Weyer, J.L., van der Weyden, L., Fikrig, E., et al. (2009). The IFITM proteins mediate cellular resistance to influenza A H1N1 virus, West Nile virus, and dengue virus. Cell *139*, 1243–1254.

Broncel, M., Serwa, R.A., Ciepla, P., Krause, E., Dallman, M.J., Magee, A.I., and Tate, E.W. (2015). Multifunctional reagents for quantitative proteome-wide analysis of protein modification in human cells and dynamic profiling of protein lipidation during vertebrate development. Angew. Chem. Int. Ed. Engl. *54*, 5948–5951.

Bruss, V., Hagelstein, J., Gerhardt, E., and Galle, P.R. (1996). Myristylation of the large surface protein is required for hepatitis B virus in vitro infectivity. Virology *218*, 396–399.

Chiu, Y.F., Sugden, B., Chang, P.J., Chen, L.W., Lin, Y.J., Lan, Y.C., Lai, C.H., Liou, J.Y., Liu, S.T., and Hung, C.H. (2012). Characterization and intracellular trafficking of Epstein-Barr virus BBLF1, a protein involved in virion maturation. J. Virol. *86*, 9647–9655.

Ciepla, P., Konitsiotis, A.D., Serwa, R.A., Masumoto, N., Leong, W.P., Dallman, M.J., Magee, A.I., and Tate, E.W. (2014). New chemical probes targeting cholesterylation of Sonic Hedgehog in human cells and zebrafish. Chem. Sci. *5*, 4249–4259.

Cox, J., and Mann, M. (2008). MaxQuant enables high peptide identification rates, individualized p.p.b.-range mass accuracies and proteome-wide protein quantification. Nat. Biotechnol. *26*, 1367–1372.

Cox, J., Neuhauser, N., Michalski, A., Scheltema, R.A., Olsen, J.V., and Mann, M. (2011). Andromeda: a peptide search engine integrated into the MaxQuant environment. J. Proteome Res. *10*, 1794–1805.

Everly, D.N., Jr., Feng, P., Mian, I.S., and Read, G.S. (2002). mRNA degradation by the virion host shutoff (Vhs) protein of herpes simplex virus: genetic and biochemical evidence that Vhs is a nuclease. J. Virol. *76*, 8560–8571.

Forrester, M.T., Hess, D.T., Thompson, J.W., Hultman, R., Moseley, M.A., Stamler, J.S., and Casey, P.J. (2011). Site-specific analysis of protein S-acylation by resin-assisted capture. J. Lipid Res. *52*, 393–398.

Frearson, J.A., Brand, S., McElroy, S.P., Cleghorn, L.A.T., Smid, O., Stojanovski, L., Price, H.P., Guther, M.L.S., Torrie, L.S., Robinson, D.A., et al. (2010). N-myristoyltransferase inhibitors as new leads to treat sleeping sickness. Nature *464*, 728–732.

Geiger, T., Wisniewski, J.R., Cox, J., Zanivan, S., Kruger, M., Ishihama, Y., and Mann, M. (2011). Use of stable isotope labeling by amino acids in cell culture as a spike-in standard in quantitative proteomics. Nat. Protoc. *6*, 147–157.

Goncalves, V., Brannigan, J.A., Thinon, E., Olaleye, T.O., Serwa, R., Lanzarone, S., Wilkinson, A.J., Tate, E.W., and Leatherbarrow, R.J. (2012). A fluorescence-based assay for N-myristoyltransferase activity. Anal. Biochem. *421*, 342–344.

Gripon, P., Le Seyec, J., Rumin, S., and Guguen-Guillouzo, C. (1995). Myristylation of the hepatitis B virus large surface protein is essential for viral infectivity. Virology *213*, 292–299.

Heal, W.P., Wright, M.H., Thinon, E., and Tate, E.W. (2012). Multifunctional protein labeling via enzymatic N-terminal tagging and elaboration by click chemistry. Nat. Protoc. *7*, 105–117.

Huang, D.W., Sherman, B.T., and Lempicki, R.A. (2009). Systematic and integrative analysis of large gene lists using DAVID bioinformatics resources. Nat. Protoc. *4*, 44–57.

Iwanaga, T., Tsutsumi, R., Noritake, J., Fukata, Y., and Fukata, M. (2009). Dynamic protein palmitoylation in cellular signaling. Prog. Lipid Res. *48*, 117–127.

Kabouridis, P.S., Magee, A.I., and Ley, S.C. (1997). S-acylation of LCK protein tyrosine kinase is essential for its signalling function in T lymphocytes. EMBO J. *16*, 4983–4998.

Konitsiotis, A.D., Jovanovic, B., Ciepla, P., Spitaler, M., Lanyon-Hogg, T., Tate, E.W., and Magee, A.I. (2015). Topological analysis of hedgehog acyltransferase, a multipalmitoylated transmembrane protein. J. Biol. Chem. *290*, 3293–3307.

Loomis, J.S., Bowzard, J.B., Courtney, R.J., and Wills, J.W. (2001). Intracellular trafficking of the UL11 tegument protein of herpes simplex virus type 1. J. Virol. *75*, 12209–12219.

MacLean, C.A., Clark, B., and McGeoch, D.J. (1989). Gene UL11 of herpes simplex virus type 1 encodes a virion protein which is myristylated. J. Gen. Virol. *70*, 3147–3157.

Macrae, D.R., Bruss, V., and Ganem, D. (1991). Myristylation of a duck hepatitis B virus envelope protein is essential for infectivity but not for virus assembly. Virology *181*, 359–363.

Martin, B.R., and Cravatt, B.F. (2009). Large-scale profiling of protein palmitoylation in mammalian cells. Nat. Methods *6*, 135–138.

Martin, B.R., Wang, C., Adibekian, A., Tully, S.E., and Cravatt, B.F. (2012). Global profiling of dynamic protein palmitoylation. Nat. Methods *9*, 84–89.

Milne, S.B., Tallman, K.A., Serwa, R., Rouzer, C.A., Armstrong, M.D., Marnett, L.J., Lukehart, C.M., Porter, N.A., and Brown, H.A. (2010). Capture and release of alkyne-derivatized glycerophospholipids using cobalt chemistry. Nat. Chem. Biol. *6*, 205–207.

Nozawa, N., Daikoku, T., Koshizuka, T., Yamauchi, Y., Yoshikawa, T., and Nishiyama, Y. (2003). Subcellular localization of herpes simplex virus type 1 UL51 protein and role of palmitoylation in Golgi apparatus targeting. J. Virol. *77*, 3204–3216.

Ohno, Y., Kashio, A., Ogata, R., Ishitomi, A., Yamazaki, Y., and Kihara, A. (2012). Analysis of substrate specificity of human DHHC protein acyltransferases using a yeast expression system. Mol. Biol. Cell *23*, 4543–4551.

Ong, S.E., Blagoev, B., Kratchmarova, I., Kristensen, D.B., Steen, H., Pandey, A., and Mann, M. (2002). Stable isotope labeling by amino acids in cell culture, SILAC, as a simple and accurate approach to expression proteomics. Mol. Cell. Proteomics *1*, 376–386.

Read, G.S., and Frenkel, N. (1983). Herpes simplex virus mutants defective in the virion-associated shutoff of host polypeptide synthesis and exhibiting abnormal synthesis of alpha (immediate early) viral polypeptides. J. Virol. *46*, 498–512.

Resh, M.D. (2004). A myristoyl switch regulates membrane binding of HIV-1 Gag. Proc. Natl. Acad. Sci. USA *101*, 417–418.

Resh, M.D. (2012). Targeting protein lipidation in disease. Trends Mol. Med. *18*, 206–214.

Rostovtsev, V.V., Green, L.G., Fokin, V.V., and Sharpless, K.B. (2002). A stepwise huisgen cycloaddition process: copper(I)-catalyzed regioselective ''ligation'' of azides and terminal alkynes. Angew. Chem. Int. Ed. Engl. *41*, 2596–2599.

Sanchez, V., Sztul, E., and Britt, W.J. (2000). Human cytomegalovirus pp28 (UL99) localizes to a cytoplasmic compartment which overlaps the endoplasmic reticulum-golgi-intermediate compartment. J. Virol. *74*, 3842–3851.

Storck, E.M., Serwa, R.A., and Tate, E.W. (2013). Chemical proteomics: a powerful tool for exploring protein lipidation. Biochem. Soc. Trans. *41*, 56–61.

Suzutani, T., Nagamine, M., Shibaki, T., Ogasawara, M., Yoshida, I., Daikoku, T., Nishiyama, Y., and Azuma, M. (2000). The role of the UL41 gene of herpes simplex virus type 1 in evasion of non-specific host defence mechanisms during primary infection. J. Gen. Virol. *81*, 1763–1771.

Tate, E.W., Kalesh, K.A., Lanyon-Hogg, T., Storck, E.M., and Thinon, E. (2015). Global profiling of protein lipidation using chemical proteomic technologies. Curr. Opin. Chem. Biol. *24*, 48–57.

Thinon, E., Serwa, R.A., Broncel, M., Brannigan, J.A., Brassat, U., Wright, M.H., Heal, W.P., Wilkinson, A.J., Mann, D.J., and Tate, E.W. (2014). Global profiling of co- and post-translationally N-myristoylated proteomes in human cells. Nat. Commun. *5*, 4919.

Tornoe, C.W., Christensen, C., and Meldal, M. (2002). Peptidotriazoles on solid phase: [1,2,3]-triazoles by regiospecific copper(I)-catalyzed 1,3-dipolar cycloadditions of terminal alkynes to azides. J. Org. Chem. *67*, 3057–3064.

Wilson, J.P., Raghavan, A.S., Yang, Y.Y., Charron, G., and Hang, H.C. (2011). Proteomic analysis of fatty-acylated proteins in mammalian cells with chemical reporters reveals S-acylation of histone H3 variants. Mol. Cell. Proteomics *10*, http://dx.doi.org/10.1074/mcp.M110.001198, M110.001198.

Wright, M.H., Pappe, D., Storck, E.M., Serwa, R.A., Smith, D.F., and Tate, E.W. (2015). Global analysis of protein N-myristoylation and exploration of N-myristoyltransferase as a drug target in the neglected human pathogen *Leishmania donovani*. Chem. Biol. *22*, 342–354.

Yasuda, K., Kosugi, A., Hayashi, F., Saitoh, S., Nagafuku, M., Mori, Y., Ogata, M., and Hamaoka, T. (2000). Serine 6 of Lck tyrosine kinase: a critical site for Lck myristoylation, membrane localization, and function in T lymphocytes. J. Immunol. *165*, 3226–3231.

Yount, J.S., Moltedo, B., Yang, Y.Y., Charron, G., Moran, T.M., Lopez, C.B., and Hang, H.C. (2010). Palmitoylome profiling reveals S-palmitoylation-dependent antiviral activity of IFITM3. Nat. Chem. Biol. *6*, 610–614.

Yurchak, L.K., and Sefton, B.M. (1995). Palmitoylation of either Cys-3 or Cys-5 is required for the biological activity of the Lck tyrosine protein kinase. Mol. Cell. Biol. *15*, 6914–6922.

Chemistry & Biology, Volume *22*

Supplemental Information

Systems Analysis of Protein Fatty Acylation in Herpes Simplex Virus-Infected Cells Using Chemical Proteomics

Remigiusz A. Serwa, Fernando Abaitua, Eberhard Krause, Edward W. Tate, and Peter O'Hare

Supplemental Information

Supplemental Figures and Tables

Supplemental Figures

Figure S1: Metabolic tagging in uninfected RPE-1 cells with fatty acid probes (YnMyr and YnPal). Relates to Figures 2.

Figure S2: HSV infection-dependent relative palmitoylation levels cross-compared to Chx treatment-dependent relative palmitoylation levels. Relates to SI Table 6.

Figure S3: Detection of HSV 1 [17] (MOI = 5) proteins metabolically tagged in RPE-1 cells with YnPal and YnMyr. Relates to SI Table 7.

Supplemental Tables

Supplementary tables have been incorporated in separate Microsoft Excel documents.

SI Table 1 HSV induced changes to protein palmitoyltion. List of 1292 proteins quantified in lysates obtained from metabolic tagging with YnPal, ligation to AzTB, and affinity enrichment. Relates to Figure 3.

SI Table 2 HSV induced changes to protein myristoylation. List of 208 proteins containing N-terminal glycine (myristoylation motif) quantified in lysates obtained from metabolic tagging with YnMyr and affinity enrichment. Relates to Figure 3.

SI Table 3 HSV induced changes to total protein abundances. List of 1428 proteins quantified in lysates obtained from metabolic tagging experiments (with YnMyr and YnPal) prior to affinity enrichment. Relates to Figure 3.

SI Table 4 HSV induced changes to nascent protein abundances. List of 510 proteins quantified in lysates obtained from 'Pulse SILAC' metabolic tagging experiments (with YnMyr) prior to affinity enrichment. At the bottom is given a list of 15 protein containing myristoylation motif (N-terminal glycine) quantified in lysates obtained from 'Pulse SILAC' metabolic YnMyr tagging experiments followed by ligation to AzTB and affinity enrichment. Relates to Figure 4.

SI Table 5 Chx induced changes to protein palmitoylation and total protein abundances. List of 496 proteins quantified from metabolic tagging with YnPal in the presence or absence of cycloheximide after ligation to AzTB, and affinity enrichment (palmitoylated). Relates to SI Figure 2.

SI Table 6 Cross comparison of the effects of HSV and Chx on protein palmitoylation. HSV infection-dependent relative palmitoylation levels correlated to Chx treatment-dependent relative palmitoylation levels in a 2-sample test. Relates to SI Figure 2.

SI Table 7 Fatty acylation of HSV encoded proteins. List of 56 HSV encoded proteins quantified in lysates obtained from metabolic tagging with YnMyr/Myr and YnPal/Pal probe pairs following ligation to AzTB and affinity enrichment. Relates to Figure 5.

SI Table 8 YnMyr tagging of HSV proteins in response to chemical inhibition of myristoylation. List of 9 HSV proteins that possess N-myristoylation motif (N-terminal glycine) quantified in lysates obtained from metabolic tagging with YnMyr in the presence and absence of an N-myristoyltransferase inhibitor. Relates to Figure 5.

SI Table 9 Functional annotation chart for proteins with decreased levels of palmitoylation. Results of functional annotation analysis performed using David Bioinformatics Resource on the set of 116 human proteins. Relates to Figure 3b and SI Table1.

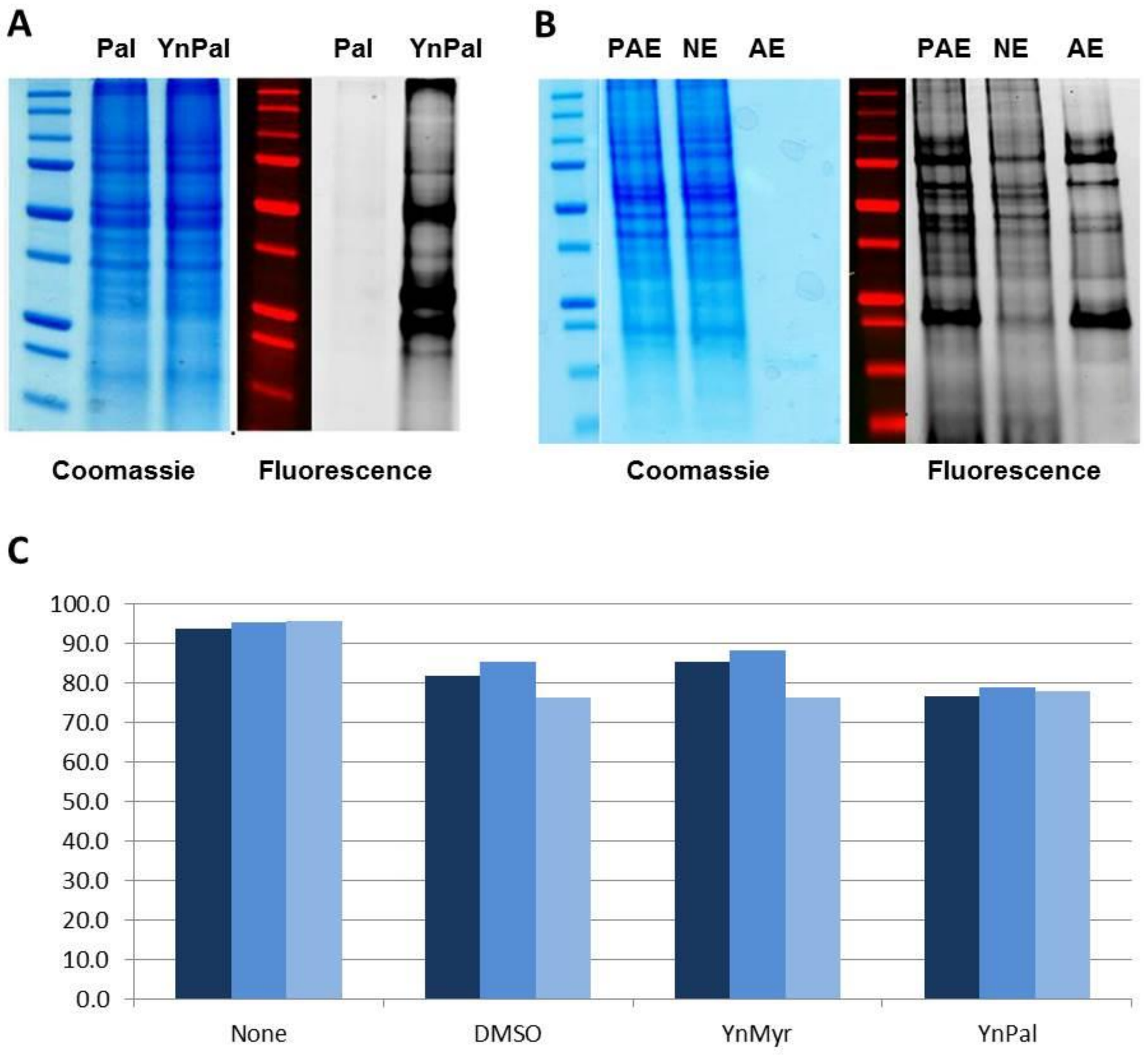

SI Figure 1. Metabolic tagging in uninfected RPE-1 cells with fatty acid probes (YnMyr and YnPal). A. RPE-1 cells were incubated with YnPal (25 µM) or with palmitic acid, Pal (25 µM) for 24 h. Cells were lysed, proteins in lysates reacted with AzTB and resolved by SDS-PAGE. Fluorescence of the gel was recorder prior to Coomassie staining (loading control). B. RPE-1 cells were incubated with YnMyr (25 µM) for 24 h. Cells were lysed, proteins reacted with AzTB and affinity enriched (streptavidin beads). Three protein fractions were applied to SDS-PAGE: AzTB reacted proteins prior to affinity enrichment (PAE); affinity enrichment (AE) and non-enriched (NE). Fluorescence of the gel was recorder prior to Coomassie staining. C. RPE-1 cells were incubated in the absence of additives (None) or in the presence of DMSO vehicle (0.1% v/v), YnMyr (25 µM), and YnPal (25 µM) for 24 h followed by viable cell counting using an automated cell counter (Countess by Invitrogen). Shades of blue indicate experimental series (n = 3).

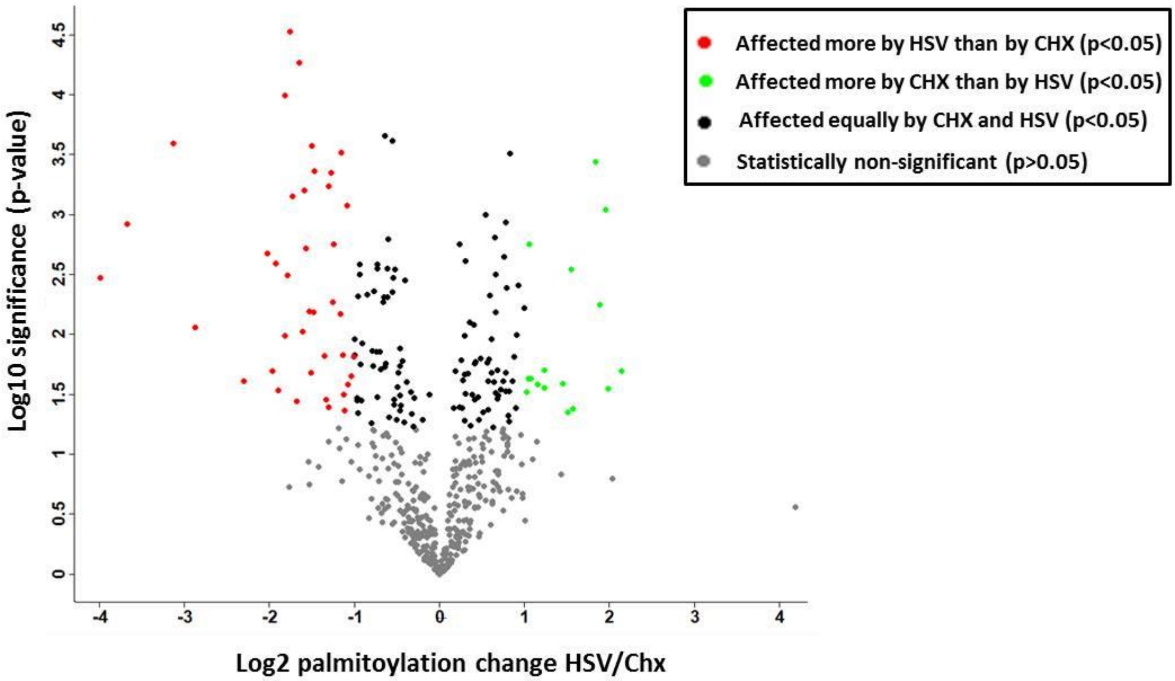

SI Figure 2. HSV infection-dependent relative palmitoylation levels cross-compared to Chx treatment-dependent relative palmitoylation levels (two-sample test, permutation based FDR = 0.05). Color-coded are different classes of proteins labelled with YnPal: proteins of which palmitoylation is affected more by HSV (red); proteins of which palmitoylation is affected more by Chx (green); proteins of which palmitoylation is affected to similar extent by HSV and Chx (black); proteins which could not be unequivocally included into the aforementioned groups due to relatively large variance within at least one of the experimental groups tested (grey). Protein identities are given in SI Table 6.

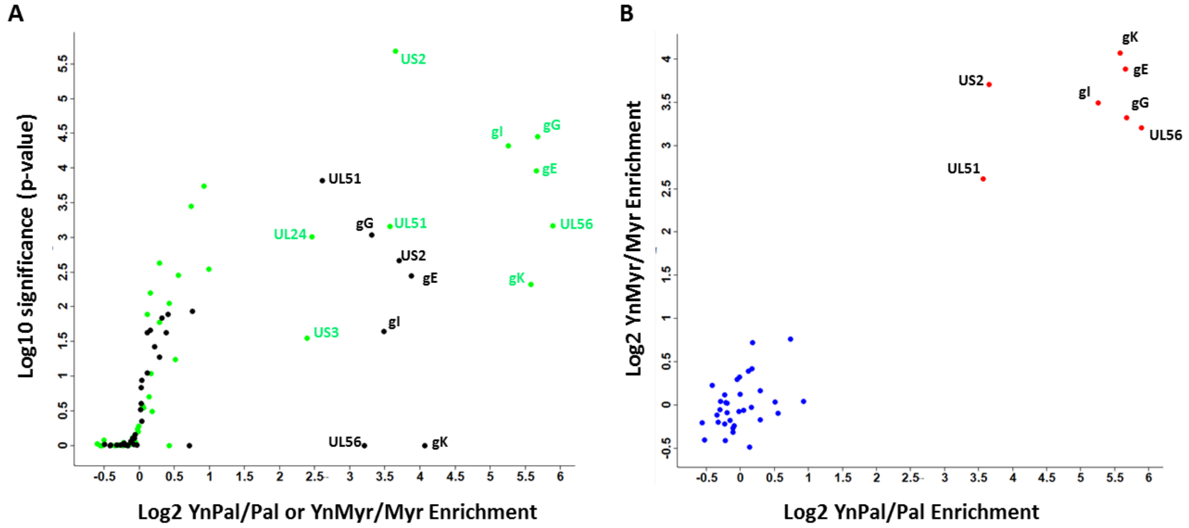

SI Figure 3. Detection of HSV 1 [17] (MOI = 5) proteins metabolically tagged in RPE-1 cells with YnPal and YnMyr. A. Palmitoylated (green) and Myristoylated (black) proteins identified on the basis of the relative enrichment from tagging with YnPal/Pal (5-19 hpi) and YnMyr/Myr (5-9 hpi), respectively. Enrichment (x-axis) plotted against significance (y-axis). B. Overlap of average YnPal/Pal and YnMyr/Myr enrichment values. Lipidated HSV protein candidates detected in both analyses are indicated in red amongst non-lipidated HSV proteins (blue).
